# Evidence of a deep viral host switch event with *beak and feather disease virus* infection in rainbow bee-eaters (*Merops ornatus*)

**DOI:** 10.1038/srep14511

**Published:** 2015-09-28

**Authors:** Subir Sarker, Kathy G. Moylan, Seyed A. Ghorashi, Jade K. Forwood, Andrew Peters, Shane R. Raidal

**Affiliations:** 1 Faculty of Science, Charles Sturt University, New South Wales 2678, Australia; 2 Alice Springs Desert Park (K. Moylan), Alice Springs, Northern Territory, 0871, Australia

## Abstract

Since the characterization of psittacine beak and feather disease (PBFD) in 1984, a wide range of avian circoviruses have been discovered with varying pathogenic effects amongst a diverse range of avian hosts. Until recently these circovirus species were thought to be restricted to within avian Orders such as the Psittaciformes for *beak and feather disease virus* (BFDV) and Columbiformes for *pigeon circovirus* with little evidence of cross-family transmission or replication. We report evidence of a naturally occurring novel host switch event with self-limiting BFDV infection in a group of rainbow bee-eaters (*Merops ornatus*) a species of Coraciiformes unrelated to parrots and not previously known to be susceptible to any avian circovirus. The outbreak highlights important and unexpected aspects of disease emergence and host-switching pertinent to other situations when viruses might cross species boundaries as well as the potential of avian circoviruses to infect disparate host species.

Circoviruses are compact circular, ambisense single-stranded DNA (ssDNA) viruses with relatively small genomes of about 2 kB^1^,^2^. A number of avian circoviruses have recently been discovered with varying pathogenic effects in a diverse range of passerine hosts including the canary (*Serinus canaria*), Gouldian finch (*Erythrura gouldiae*), common starling (*Sternus vulgaris*) and Australian raven (*Corvus coronoides*) which are all naturally susceptible to avian circovirus species^3^,^4^,^5^,^6^. Non-passerine birds such as anatids, lariids and columbids are also well represented with circovirus species found in various species of ducks, geese, swans, gulls and pigeons^7^,^8^. Pigeon, goose and canary circoviruses have all been officially classified as members of the genus *Circovirus* and they share similar genomic and structural characteristics to other circoviruses. *Pigeon circovirus* (PiCV) is distributed worldwide with infection associated with lethargy, anorexia, runting and poor racing performance as part of a multifactorial syndrome in affected pigeons and doves^8^. Similarly *goose circovirus* (GoCV) is associated with a runting syndrome and is also expected to have a worldwide distribution^9^.

Throughout Australia psittacine beak and feather disease (PBFD) caused by the circovirus *beak and feather disease virus* (BFDV) is a very common, chronic but ultimately fatal disease of Psittaciformes, characterized by long term incubation and progressive feather dystrophy^10^,^11^. Of the 350 extant parrot, lorikeet and cockatoo species, all are considered susceptible to BFDV infection^12^,^13^. BFDV is highly genetically diverse and prone to mutation but relatively antigenically conserved with little evidence of different antigenic serotypes. Whilst BFDV is subject to a high mutation rate, approaching that of RNA viruses, strong recombination pressure and regular cross-species transmission occurs throughout the Psittaciformes^14^,^15^,^16^. Whilst it has a worldwide distribution in captive birds, where high rates of transmission and a rich viral genetic diversity can occur in captivity, recent genetic analysis supports Australia as the most likely source of all global BFDV genotypes^12^,^17^,^18^. This is not surprising given the rich psittacine avifauna of the Australasian region. Recent evidence also indicates that BFDV may be able to infect non-psittacine bird species with one report of BFDV replicase gene (*Rep*) sequences in Gouldian finches that had feather lesions suggestive of PBFD^19^. Such events are presumably rare but probably more likely to occur when captive psittacine and non-psittacine bird species are mixed together in captivity. Here we report evidence of BFDV-infection in wild rainbow bee-eaters (*Merops ornatus*) from central Australia which likely represents a naturally occurring novel host switch event with evidence of self-limiting BFDV infection in a group of rainbow bee-eaters, a species of Coraciiformes unrelated to parrots and not previously known to be susceptible to circovirus infection. The outbreak highlights important and unexpected aspects of cryptic disease emergence and host-switching pertinent to other situations when viruses undergo spillover across relatively deep phylogenetic divides.

## Results

### Pathology and serology

**Figure 1:**
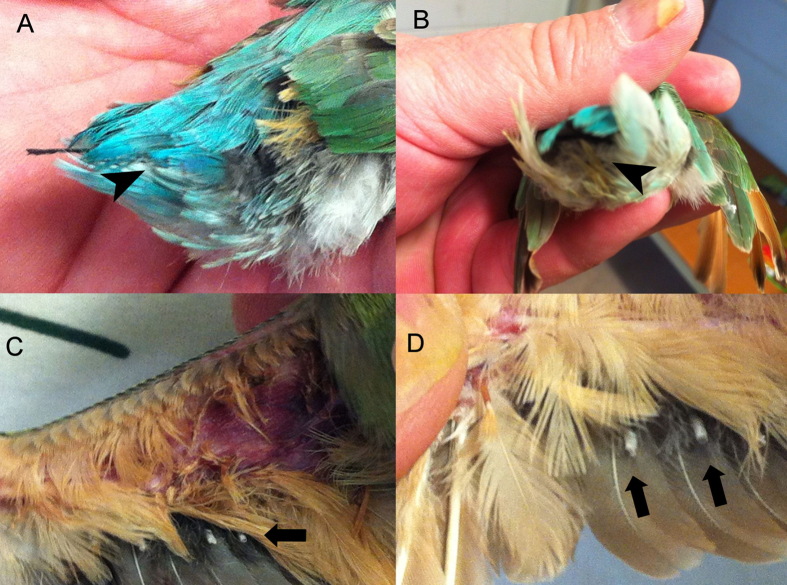
Plumage deficits in rainbow bee-eaters. There is bilaterally symmetrical loss of primary flight feathers. (**A,B**) demonstrates broken rectrices (arrow heads). (**C,D**) demonstrates broken remiges (arrows), and thickened feather sheaths akin to the lesions seen in psittacine beak and feather disease.

Rainbow bee-eaters with plumage deficits and dystrophic flight feathers similar to PBFD are shown in Fig. 1. Histological examination of multiple sections of skin and visceral organs revealed mild to moderate orthokeratotic hyperkeratosis of the epidermal structures and feather follicles with mild hyperkeratosis of feather sheaths but visceral organs appeared histologically normal. Immunohistochemistry for BFDV was negative for all tissues. HA was not detected in feather samples and anti-BFDV HI antibody was not detected in blood from any of the rainbow bee-eater samples tested.

### PCR testing

Six rainbow bee-eaters were positive for BFDV by PCR assays using circovirus degenerate primers. For the majority of birds (5 out of 6) both the DNA extracted from blood and feather material were positive. From bird 14-1186-03 (GenBank accession no. KM823547) only the feather sample was PCR positive. In two birds (14-1186-02 and 14-1186-04) more than one BFDV genotypic variants were detected using DNA sourced from blood and feather material (GenBank accession no. KM823542 and KM823546; KM823543 and KM823548 respectively). In 3 other birds identical BFDV DNA sequences were obtained from blood and feather material. PCR results were verified at least twice during the initial testing period and all birds returned PCR negative results when their plumage recovered some 6 months after initial presentation. A clinically normal mulga parrot (*Psephotus varius*) at the facility which was not in direct contact with the bee eaters was PCR negative for BFDV.

### Phylogenetic analysis of the BFDV sequences from rainbow bee-eaters

One entire genome and 7 unique partial *Rep* sequences were obtained from six infected birds for analysis. A maximum-likelihood phylogenetic tree of a single full BFDV genome (KM823541) sequenced from one of the rainbow bee-eaters (Supplementary Figure 1) showed it clustered with Australian BFDV genotypes from a wild red-tailed black cockatoo (*Calyptorhyncus banksii*, KF385399), a hooded parrot (*Psephotus dissimilis*, KF688553), a golden-shouldered parrot (*Psephotus chrysopterrygius*, KF688552) and three wild Australian ringneck parrots (*Barnardius zonarius semitorquatus*, KF688548-50). There was 82.2–95.6% pairwise nucleotide identity between the rainbow bee-eater genotype and other BFDV in GenBank with the highest similarity (95.6%) to a BFDV from a wild red-tailed black cockatoo from Western Australia (KF385399).

### Genetic diversity of BFDV amongst infected rainbow bee-eaters

**Figure 2:**
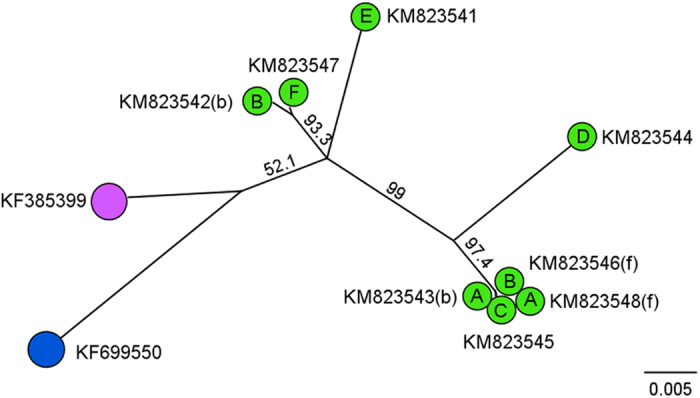
Phylogenetic inference of evolutionary relationship among *Rep* gene sequences from rainbow bee-eaters. Phylogenetic analysis of partial BFDV *Rep* sequences from rainbow bee-eaters (green) constructed using the NJ method with Tamura-Nei distance estimation, with 1000 bootstrap resamplings and BFDV from a wild red-tailed black cockatoo (purple, GenBank accession: KF385399) and a wild Australian ringneck parrot (blue, GenBank accession:KF699550) as outgroups. Individual upper case letters identify BFDV genomes from individual birds (**A** to **F**) with the source of DNA shown in brackets with (**b**) for blood and (**f**  ) for feather material.

Alignments of partial *Rep* sequences from rainbow bee-eaters demonstrated significant diversity except for sequences from four birds (GenBank accession numbers KM823543, KM823544, KM823545 and KM823548), which consisted of a single clade (Fig. 2). Two different genotypic variants (GenBank accessions KM823542 and KM823546) were from blood and feather samples, respectively, of a single rainbow bee-eater and this showed greater *Rep* genetic distance between themselves than from all other rainbow bee-eater *Rep* sequences analysed in this study (93% similarity) with predicted amino acid identity having even greater distance (76% pairwise identity).

## Discussion

This paper documents BFDV infection in rainbow bee-eaters as a presumed host switch event. Whilst corroborating evidence of infection such as BFDV antigen excretion and seroconversion were not present in the birds, they did have transient clinical signs and feather lesions consistent with all other avian circovirus diseases so far documented. Evidence of BFDV infection as a cause of the feather lesions seen in the bee-eaters was largely reliant on the multiple positive PCR blood results that were consistently obtained from different birds in the affected flock. Copious shedding of BFDV occurs from the feathers of PBFD-affected psittacine birds and the risk of environmental contamination of samples is great when PCR tests are used as the main diagnostic modality. Collection and testing of bodily tissues, such as blood and internal organs, especially the spleen, minimized this risk to a great extent^10^,^12^,^20^. Sequencing of samples confirmed that the rainbow bee-eater BFDV sequences were different from any previously sequenced BFDV genotypes. Evidence of quasispecies variation in the rainbow bee-eater samples (Supplementary Figure 2) is also best explained by *in vivo* replicative modulation as part of host adaptation but could also be the result of genetic variation within an infectious dose. Nevertheless, the findings provide strong evidence that the results obtained for the bee-eaters are indicative of naturally occurring BFDV infection, even if self-limiting.

The pathology, PCR testing and DNA sequence evidence supports the conclusion that the birds were infected in the wild before they were collected. Iatrogenic or nosocomial infection within the zoo facility is unlikely considering that the hygiene controls in place once the birds had been captured should have been sufficient to prevent in-direct transmission of infection. Whilst BFDV is likely to be a highly resilient virus based on its physicochemical properties there is no evidence that it is highly infectious even for psittacine birds which are its natural host. The early studies performed in the 1980’s showed that it was quite difficult to experimentally transmit the disease in parrots using very high concentrations of virus^21^. More recent research has shown that susceptible young corellas may become experimentally infected, detectable by PCR or seroconversion, only when given very large amounts of the virus and yet still not develop any signs of feather disease^22^. It seems that very high doses are required to establish infection but the age of birds when they are exposed is likely to have the greatest influence on the outcome of infection, with the youngest birds (aged only a few days) much more susceptible to developing disease than older nestlings or fledglings^21^.

BFDV may behave like cypovirus and baculovirus polyhedra^23^,^24^ in natural transmission events. During their replication cycle cypoviruses and baculoviruses, which are pathogens of insects, form large, micrometre-sized, virus embedded protein crystals called polyhedra which provides them with added environmental stability and long lived infectivity in soil. The intracytoplasmic inclusions of BFDV are of similar size and physicochemical characteristic, and are likely to contain a population of many genetic variants within an infective dose^12^,^15^. So the presence of quasipecies variation could reflect either genetic variation within the infective dose, or variation with abortive replication attempts, or combinations of both.

The initial negative test results using the robust PCR primer set P2–P4 which targets the Rep gene of all previously known BFDV^25^ indicates that there may be more diverse BFDV genotypes circulating or stochastically spilling over into a wider variety of bird species than previously thought. Recent research has shown that during the course of infection numerous BFDV genome variants may be present in individually infected psittacine birds alongside a high mutation rate^12^,^15^. This is probably a replication strategy that allows flexible host-switching, at least amongst psittacine birds^12^,^15^. BFDV along with other avian circoviruses has a relatively small genome of only 2.0 kb with only two genes. This simplicity exists alongside relatively constrained genome size and antigenic diversity in the face of high mutability and relatively broad genetic diversity^1^,^2^,^11^. Unlike other DNA viruses the International Committee on Taxonomy of Viruses (ICTV) criteria demarcates species in the *Circovirus* genus to less than 75% whole genome similarity, reflecting typical diversity within circovirus lineages. Such diversity permits a relatively broad sequence space for individual circovirus species to explore. These features give BFDV replication characteristics more akin to RNA viruses, which tend to more broadly have the capacity to undergo relatively frequent host-switching^12^.

In an attempt to discover any possible iatrogenic infection we analyzed and tested all possible parrot species in contact with the infected rainbow bee-eaters. The rainbow bee-eaters in our study had been raised in quarantine by a dedicated zoo-keeper with stringent hygiene control^26^ which included hand washing with chlorhexidine between handling and no direct or indirect contact with any parrots. The closest parrot, a mulga parrot, which was housed in a completely separate building proved to be PCR negative and seronegative for BFDV. The timing and source of the infection is therefore unknown but most likely to have occurred in the wild before the birds were collected. Parrots are common throughout Australia and there would have been ample opportunity for indirect exposure to BFDV in the wild.

Some aspects of this case are difficult to explain fully without conducting ethically debatable experimental virus-transmission experiments. Rainbow bee-eaters are a migratory bird that does not intimately share any ecological niche with any known parrot species. They nest by burrowing 1 m long tunnels into the sand of river beds and build new nest sites each year^27^. They are predominantly insectivorous, catching flying insects on the wing, so do not share any food resources with the four common psittacine species found in the remote region where the birds were collected. Rainbow bee-eaters are rarely seen in close contact with parrots at watering holes or at perches. A possible scenario for transmission could be ingestion of BFDV-contaminated insect vectors including hippoboscid flies such as *Ornithomyia* spp., which are common hitchhikers of parrots, or other flying haematophagous insects that cohabit active parrot nest hollows. Alternatively they may have been infected within their nest burrow since BFDV infectivity is likely to persist in the environment for many months^26^. Parrots rarely use ground burrows and there are very few extant ground-nesting parrots in Australia. The likely extinct night parrot (*Pezoporus occidentalis*), is the only ground-nesting species to have potentially existed historically within the region. Whilst Fig. 1 shows a close relationship of the outbreak with BFDV (KF385399) from a red-tailed black cockatoo it is very unlikely that this species, or cockatoos more broadly, were the source of infection given the location of this genotype within a clade of both geographically and broadly genetically diverse host species^12^. The parrots of the *Barnardius* genus are a more likely source given their broad geographic range and abundance across Australia^28^.

Viral persistence was not detected in the rainbow bee-eaters with all birds eventually returning PCR negative results and recovering from feather lesions. Failure to detect BFDV antigen by IHC in feather follicles and skin is likely to have been a result of sampling during a phase of recovery from transient infection and or incomplete BFDV replication within keratinocytes.

BFDV is a haemagglutinating virus and is able to interact with erythrocytes from a number of psittacine bird species but even within psittacine bird species BFDV haemagglutination activity is confined to the blood from individual birds^29^,^30^ which is unusual compared to other haemagglutinating viruses. The blood from some individual guinea pigs and geese are also susceptible to haemagglutination^31^,^32^ so there is evidence that BFDV can at least interact with unknown receptors on the surfaces of distantly related avian and non-avian cells.

The rainbow bee-eaters failed to develop anti-BFDV antibodies, which may have been due to low sensitivity of the assay or non-competent BFDV replication. Considering that the birds were captured from the wild the role of stress in permitting transient infection and or interfering with antibody responses cannot be ruled out. Nevertheless, many PBFD-affected birds such as cockatoos are almost always seronegative^20^,^33^ and the same situation may be the case for these rainbow bee-eaters. Low antibody response has been postulated^34^ as a marker of BFDV tolerance in crimson rosellas (*Platycercus elegans*). In the affected rainbow bee-eaters antibody levels that are absent or below the detection limit may represent a tolerance mechanism to BFDV or there may have been transient replication in immunologically restricted sites such as the feather follicle.

Due to the abundance and wide distribution of parrots in the Australian landscape and the high prevalence of BFDV infection in many of the most common species of parrots there can be little doubt that birds from other orders are exposed to BFDV frequently. This exposure would typically lead to infection with subsequent immune clearing of the virus or the development of clinical signs. The response of non-psittacine birds to such exposure however remains unknown. We can nevertheless expect from broader host-virus infection theory that the response will involve either 1) failure to establish a patent infection, 2) clearance of the virus through an appropriate immunological response or 3) failure to clear the virus. The characteristic of viral shedding is a separate consideration and will define the ability of the new host species to transmit the virus to other individuals or the environment. The absence of BFDV antigen excretion in the rainbow bee-eaters is likely indicative of failure of viral transmission in this new host species (i.e. R_0 _~ 0) which may be a result of obstruction of viral replicative pathways in the new host. It also indicates that these birds were all independently infected through a shared contaminated environmental source rather than transmission within the flock.

Of the three scenarios following exposure in a new host, the return to an absence of viraemia in the birds indicates that the virus was cleared or never established patency in the first place. The lack of an antibody response to BFDV, which appears to be robust in psittacine hosts^20^, suggests that clearance through adaptive immune mechanisms is equally unlikely, although innate immunity may certainly still have played a role. In light of these findings, the most plausible scenario is one of exposure, failure of primary defense against viral infection (possibly through stress-induced suppressed innate immunity), viraemia and the initiation of clinical signs through the epitheliotropic nature of BFDV, but with subsequent failure to complete viral replication and excretion with gradual clearance through cell turnover. Failure of competent BFDV replication can explain the histological absence of inclusions or IHC reactivity, as BFDV inclusions are a product of viral replication. This failure of BFDV to complete its life cycle is understandable considering the deep evolutionary divide between the Psittaciformes and the Coraciiformes to which rainbow bee-eaters belong. More surprising is the ability of BFDV to get as far in the new host as it did, which may in this case be simply a function of failed innate immunity and a high initial exposure. The pertinent outcomes of our findings however are that non-psittacine avian orders in Australia may be exposed to infective levels of BFDV even in the absence of clear ecological association with parrots; that BFDV may be capable of infecting non-psittacine birds; and that BFDV infection in aberrant hosts may not always lead to an adaptive immune response, viral replication and/or shedding.

## Methods

### Source of Sampling

Samples used in the analyses described below were obtained during the course of health monitoring of birds obtained and held for captive breeding by at the Alice Springs Desert Park. Animal sampling was obtained in accordance with approved guidelines set by the Australian Code of Practice for the Care and Use of Animals for Scientific Purposes (1997) and approved by the Charles Sturt University Animal Ethics Committee (Research Authority permit 09/046).

Samples from a total of nine juvenile rainbow bee-eaters (*Merops ornatus*) with feather defects were collected for circovirus detection. The birds had been collected from the wild (year of sampling 2014, GPS location, −23.70606, 133.83245) as two separate broods of nestlings for the purposes of display at a zoological park. They were aged approximately 2–3 weeks when collected. The first brood of five chicks all developed plumage deficits and dystrophic flight feathers (Fig. 1) similar to the lesions seen in PBFD within a few weeks of being captured. Some fledgling birds in a second brood of chicks, which had been similarly taken from the wild and housed together with the first group of birds, also developed flight feather lesions of similar severity with others developing poor contour feather development. Two chicks with subtle feather lesions and poor weight gain were euthanazed and histopathological examination of the visceral organs and skin performed. Sections of skin, spleen and visceral organs were tested by immunohistochemistry for BFDV according to established protocols^35^.

During the initial sampling to investigate the feather lesions and in an effort to detect seroconversion the birds were sampled one week apart. To do this blood was collected from each bird onto filter paper and fragments of broken feathers and developing feathers plucked from follicles were collected for serology and avian circovirus detection by PCR, respectively. All birds were then retested six months later, with blood and feather samples collected from all nine affected fledglings. Furthermore, a blood sample was collected from a mulga parrot (*Psephotus varius*) which was the only captive parrot within in-direct contact in an attempt to discover potential sources of BFDV infection.

### Haemagglutination (HA) and Haemagglutination inhibition (HI) assays

In an effort to detect BFDV antigen excretion in feather dander HA assays were performed on feather samples using established protocols^20^. Similarly HI assays were done to detect evidence of anti-BFDV antibody in blood samples collected onto filter paper^20^.

### Amplification of DNA by nested PCR

Initially, BFDV DNA extracted from feather and blood samples^36^ was screened by an established PCR protocol that targets 717-bp section of the BFDV *Rep*^25^. When samples from all birds except one were detected negative by this PCR protocol a degenerative avian circovirus primer set and protocol was used as outlined below. Published circovirus genome sequences were retrieved from the literature and NCBI GenBank^37^ and aligned with Geneious Pro 6.1.8 for designing primers. The *Rep* was chosen for amplification and two sets of degenerate primers were designed. The first-round PCR using the primer set FWD CV-s (5′-AGAGGTGGGTCTTCACNHTBAAYAA-3′) and REV CV-as (5′-AAGGCAGCCACCCRTARAARTCRTC-3′) was optimized to contain 2 μL of extracted genomic DNA, 25 pM of each primer (GeneWorks, Australia), 1.5 mM MgCl_2,_ 1.25 mM of each dNTP, 1×GoTaq^®^ Green Flexi Reaction Buffer, 1 U of Go Taq DNA polymerase (Promega Corporation, USA) and DEPC distilled H_2_O (Invitrogen, USA) was added in a final volume of 25 μL. The first round amplification was carried out in an iCycler thermal cycler (Bio-Rad) under the following conditions: denaturation at 95 °C for 5 min followed by 40 cycles of 95 °C for 30 s, 51 °C for 30 s and 72 °C for 1 min, and a final extension step of 10 min at 72 °C.

The nested PCR using the primer set FWD Cn-s (5′-AGCAAGGAACCCCTCAYYTBCARGG-3′) and REV Cn-as (5′-ACGATGACTTCNGTCTTSMARTCACG-3′) was performed in a 25 μL reaction volume on an iCycler thermal cycler (Bio-Rad). The master mix for nested PCR was the same as that of the first-round PCR, except it contained 25 pM of each primer FWD Cn-s and REV Cn-as and 2 μL of previous PCR product. Thermal cycling was carried out as described for first-round PCR, except that the annealing temperature was set to 52 °C.

An established protocol for the amplification of BFDV genome^12^,^15^ was modified with additional primers based on partial sequences obtained from rainbow bee-eaters (BFDV-K-R; 5′-ACGGCCTTGCCTTCAGCT-3′, and BFDV-C-F; 5′-ACTATGCCATCGTTGGACG-3′) which were designed and used to facilitate sequencing of the entire genome. Amplified PCR products, together with a standard molecular mass marker (Sigma), were separated by electrophoresis in 0.8% agarose gel stained with GelRed (Biotium, CA) using 0.5× TBE buffer at 90 V for 1.5 h, and the appropriate bands were excised and purified using the Wizard^®^ SV Gel and PCR Clean-Up System (Promega, USA) according to the manufacturer’s instructions. Purified amplicons were sequenced with PCR and internal primers by the Australian Genome Research Facility Ltd (Sydney) using an AB 3730xl unit (Applied Biosystems). For each amplicon sequences were obtained at least twice in each direction for each isolate to ensure the validity of the data. The sequences were trimmed for primers, aligned to construct contigs using a minimum overlap of 35 bp and a minimum match percentage of 95%, and construction of full genome and partial sequences were carried out in Geneious Pro and BioEdit Sequence Alignment Editor (version 7.1.6.0).

### Phylogenetic analyses

A genome sequence of BFDV determined in this study was aligned with all known BFDV sequence data available on the literature and NCBI GenBank (supplementary Table S1) using the MAFFT L-INS-i algorithm (scoring matrix = 200PAM/K = 2; gap open penalty = 1.53)^38^ in Geneious. Multiple sequence alignments of the complete BFDV genome, as well as partial *Rep* sequences from rainbow bee-eaters were used to determine the phylogenetic relationships of the viruses. The program jModelTest 2.1.3 favoured a general-time-reversible model with gamma distribution rate variation and a proportion of invariable sites (GTR +I +G4) for the Maximum likelihood (ML) analysis^39^. ML and Neighbour-joining (NJ) phylogenetic trees were rooted with *raven circovirus*^4^, and 1,000 non-parametric bootstrap resamplings were generated using Geneious software.

## Additional Information

**How to cite this article**: Sarker, S. *et al.* Evidence of a deep viral host switch event with *beak and feather disease virus* infection in rainbow bee-eaters (*Merops ornatus*). *Sci. Rep.* **5**, 14511; doi: 10.1038/srep14511 (2015).

## Supplementary Material

Supplementary Information

## References

[b1] Ritchie B. *et al.* Ultrastructural, protein composition, and antigenic comparison of psittacine beak and feather disease virus purified from four genera of psittacine birds. J Wildl Dis. 26, 196–203 (1990). 2338723 10.7589/0090-3558-26.2.196

[b2] Bassami M. R., Ypelaar I., Berryman D., Wilcox G. E. & Raidal S. R. Genetic diversity of beak and feather disease virus detected in psittacine species in Australia. Virology 279, 392–400 (2001). 11162795 10.1006/viro.2000.0847

[b3] Todd D. *et al.* Molecular characterization of novel circoviruses from finch and gull. Avian Pathol. 36, 75–81 (2007). 10.1080/03079450601113654 17364513

[b4] Stewart M. E., Perry R. & Raidal S. R. Identification of a novel circovirus in Australian ravens (*Corvus coronoides*) with feather disease. Avian Pathol. 35, 86–92 (2006). 10.1080/03079450600597345 16595298

[b5] Johne R., Fernández-de-Luco D., Höfle U. & Müller H. Genome of a novel circovirus of starlings, amplified by multiply primed rolling-circle amplification. J Gen Virol. 87, 1189–1195 (2006). 10.1099/vir.0.81561-0 16603520

[b6] Phenix K. V. *et al.* Nucleotide sequence analysis of a novel circovirus of canaries and its relationship to other members of the genus Circovirus of the family Circoviridae. J Gen Virol. 82, 2805–2809 (2001). 11602793 10.1099/0022-1317-82-11-2805

[b7] Scott A. N. J. *et al.* Serological diagnosis of goose circovirus infections. Avian Pathology 35, 495–U420 (2006). 10.1080/03079450601087841 17121739

[b8] Duchatel J. P., Todd D., Smyth J. A., Bustin J. C. & Vindevogel H. Observations on detection, excretion and transmission of pigeon circovirus in adult, young and embryonic pigeons. Avian Pathology 35, 30–34 (2006). 10.1080/03079450500465692 16448939

[b9] Todd D., Weston J. H., Soike D. & Smyth J. A. Genome sequence determinations and analyses of novel circoviruses from goose and pigeon. Virology 286, 354–362 (2001). 10.1006/viro.2001.0985 11485403

[b10] Raidal S. R., McElnea C. L. & Cross G. M. Seroprevalence of psittacine beak and feather disease in wild psittacine birds in New South Wales. Aust Vet J. 70, 137–139 (1993). 10.1111/j.1751-0813.1993.tb06105.x 8494522

[b11] Ritchie B. W., Niagro F. D., Lukert P. D., Steffens Iii W. L. & Latimer K. S. Characterization of a new virus from cockatoos with psittacine beak and feather disease. Virology 171, 83–88 (1989). 10.1016/0042-6822(89)90513-8 2741350

[b12] Sarker S. *et al.* Phylogeny of beak and feather disease virus in cockatoos demonstrates host generalism and multiple-variant infections within Psittaciformes. Virology 460-461, 72–82 (2014). 10.1016/j.virol.2014.04.021 25010272

[b13] Peters A. *et al.* Evidence of Psittacine Beak and Feather Disease Virus Spillover into Wild Critically Endangered Orange-Bellied Parrots (*Neophema chrysogaster*). J Wildl Dis. 50, 288–296 (2014). 10.7589/2013-05-121 24484492

[b14] Julian L. *et al.* Extensive recombination detected amongst Beak and feather disease virus isolates from breeding facilities in Poland. J Gen Virol. 94, 1086–1095 (2013). 10.1099/vir.0.050179-0 23324468

[b15] Sarker S. *et al.* Mutability dynamics of an emergent single stranded DNA virus in a naïve host. PLoS ONE 9, e85370 (2014). 10.1371/journal.pone.0085370 24416396 PMC3885698

[b16] Kundu S. *et al.* Tracking Viral Evolution during a Disease Outbreak: the Rapid and Complete Selective Sweep of a Circovirus in the Endangered Echo Parakeet. J Virol. 86, 5221–5229 (2012). 10.1128/jvi.06504-11 22345474 PMC3347377

[b17] Sarker S., Ghorashi S. A., Forwood J. K. & Raidal S. R. Rapid genotyping of beak and feather disease virus using high-resolution DNA melt curve analysis. J Virol Methods 208, 47–55 (2014). 10.1016/j.jviromet.2014.07.031 25102431

[b18] Harkins G. W., Martin D. P., Christoffels A. & Varsani A. Towards inferring the global movement of beak and feather disease virus. Virology 450, 24–33 (2014). 10.1016/j.virol.2013.11.033 24503064

[b19] Circella E. *et al.* Psittacine Beak and Feather Disease–like Illness in Gouldian Finches (*Chloebia gouldiae*). Avian Dis. 58, 482–487 (2014). 10.1637/10745-121113Case.1 25518446

[b20] Raidal S. R., Sabine M. & Cross G. M. Laboratory diagnosis of psittacine beak and feather disease by haemagglutination and haemagglutination inhibition. Aust Vet J. 70, 133–137 (1993). 8494521 10.1111/j.1751-0813.1993.tb06104.x

[b21] Wylie S. L. & Pass D. A. Experimental reproduction of psittacine beak and feather disease/French Moult. Avian Pathol. 16, 269–281 (1987). 18766613 10.1080/03079458708436374

[b22] Bonne N., Shearer P., Sharp M., Clark P. & Raidal S. Assessment of recombinant beak and feather disease virus capsid protein as a vaccine for psittacine beak and feather disease. J Gen Virol. 90, 640–647 (2009). 10.1099/vir.0.006932-0 19218209

[b23] Coulibaly F. *et al.* The atomic structure of baculovirus polyhedra reveals the independent emergence of infectious crystals in DNA and RNA viruses. Proc Natl Acad Sci USA. 106, 22205–22210 (2009). 10.1073/pnas.0910686106 20007786 PMC2799703

[b24] Coulibaly F. *et al.* The molecular organization of cypovirus polyhedra. Nature 446, 97–101 (2007). 10.1038/nature05628 17330045

[b25] Ypelaar I., Bassami M. R., Wilcox G. E. & Raidal S. R. A universal polymerase chain reaction for the detection of psittacine beak and feather disease virus. Vet Microbiol. 68, 141–148 (1999). 10501171 10.1016/s0378-1135(99)00070-x

[b26] Cross G. M. *Hygiene Protocols for the Prevention and Control of Diseases (Particularly Beak and Feather Disease) in Australian Birds.* Department of Sustainability Environment Water Population and Communities. (2006). http://www.environment.gov.au/node/16197 Date of access: 17/12/2014.

[b27] Boland C. R. J. Breeding biology of Rainbow Bee-Eaters (*Merops ornatus*): A migratory, colonial, cooperative bird. Auk 121, 811–823 (2004).

[b28] Das S., Sarker S., Forwood J. K., Ghorashi S. A. & Raidal S. R. Characterization of the Whole-Genome Sequence of a Beak and Feather Disease Virus Isolate from a Mallee Ringneck Parrot (*Barnardius zonarius barnardi*). Genome Announc 2, (2014). 10.1128/genomeA.00708-14 PMC4110224 25059866

[b29] Sanada N. & Sanada Y. The sensitivities of various erythrocytes in a haemagglutination assay for the detection of psittacine beak and feather disease virus. J Vet Med Series B-Infect Dis Vet Pub Health 47, 441–443 (2000). 10.1046/j.1439-0450.2000.00360.x 11014065

[b30] Raidal S. R. & Cross G. M. The hemagglutination spectrum of psittacine beak and feather disease virus. Avian Pathol 23, 621–630 (1994). 18671129 10.1080/03079459408419032

[b31] Sexton N., Penhale W. J., Plant S. L. & Pass D. A. Use of goose red blood cells for detection of infection with psittacine beak and feather disease virus by haemagglutination and haemagglutination inhibition. Aust Vet J. 71, 345–347 (1994). 7848185 10.1111/j.1751-0813.1994.tb00917.x

[b32] Ritchie B. W. *et al.* Hemagglutination by psittacine beak and feather disease virus and use of hemagglutination inhibition for detection of antibodies against the virus. Am J Vet Res. 52, 1810–1815 (1991). 1785723

[b33] Khalesi B., Bonne N., Stewart M., Sharp M. & Raidal S. A comparison of haemagglutination, haemagglutination inhibition and PCR for the detection of psittacine beak and feather disease virus infection and a comparison of isolates obtained from loriids. J Gen Virol. 86, 3039–3046 (2005). 10.1099/vir.0.81275-0 16227226

[b34] Eastwood J. R. *et al.* Phylogenetic analysis of beak and feather disease virus across a host ring-species complex. Proc Natl Acad Sci USA. 111, 14153–14158 (2014). 10.1073/pnas.1403255111 25225394 PMC4191811

[b35] Shearer P. L., Bonne N., Clark P., Sharp M. & Raidal S. R. Development and applications of a monoclonal antibody to a recombinant beak and feather disease virus (BFDV) capsid protein. J. Virol. Methods 147, 206–212 (2008). 10.1016/j.jviromet.2007.08.029 17942162

[b36] Bonne N., Clark P., Shearer P. & Raidal S. Elimination of false-positive polymerase chain reaction results resulting from hole punch carryover contamination. J Vet Diagn Invest. 20, 60–63, (2008). 10.1177/104063870802000111 18182510

[b37] Benson D. A. *et al.* GenBank. Nucleic Acids Res. 41, D36–42 (2013). 10.1093/nar/gks1195. 23193287 PMC3531190

[b38] Katoh K., Misawa K., Kuma K. i. & Miyata T. MAFFT: a novel method for rapid multiple sequence alignment based on fast Fourier transform. Nucleic Acids Res. 30, 3059–3066 (2002). 10.1093/nar/gkf436 12136088 PMC135756

[b39] Darriba D., Taboada G. L., Doallo R. & Posada D. jModelTest 2: more models, new heuristics and parallel computing. Nat Meth 9, 772 (2012). 10.1038/nmeth.2109 PMC4594756 22847109

