# Supplementary material for: Evidence of a deep viral host switch event with beak and feather disease virus infection in rainbow bee-eaters (Merops ornatus)
Source: Sci Rep. 2015 Sep 28;5:14511. doi: 10.1038/srep14511 (PMC4585972; doi:10.1038/srep14511)
Supplement: Supplementary Information [file srep14511-s1.pdf]

SUPPLEMENTARY INFORMATION

Evidence of a deep viral host switch event with *beak and feather disease virus* infection in rainbow bee-eaters (*Merops ornatus*)

Subir Sarker,[1] Kathy G. Moylan,[2] Seyed A. Ghorashi, [1] Jade K. Forwood, [1] Andrew Peters[1] and Shane R. Raidal[1]

[1]Faculty of Science, Charles Sturt University, New South Wales 2678, Australia

[2]Alice Springs Desert Park (K. Moylan), Alice Springs, Northern Territory, 0871, Australia.

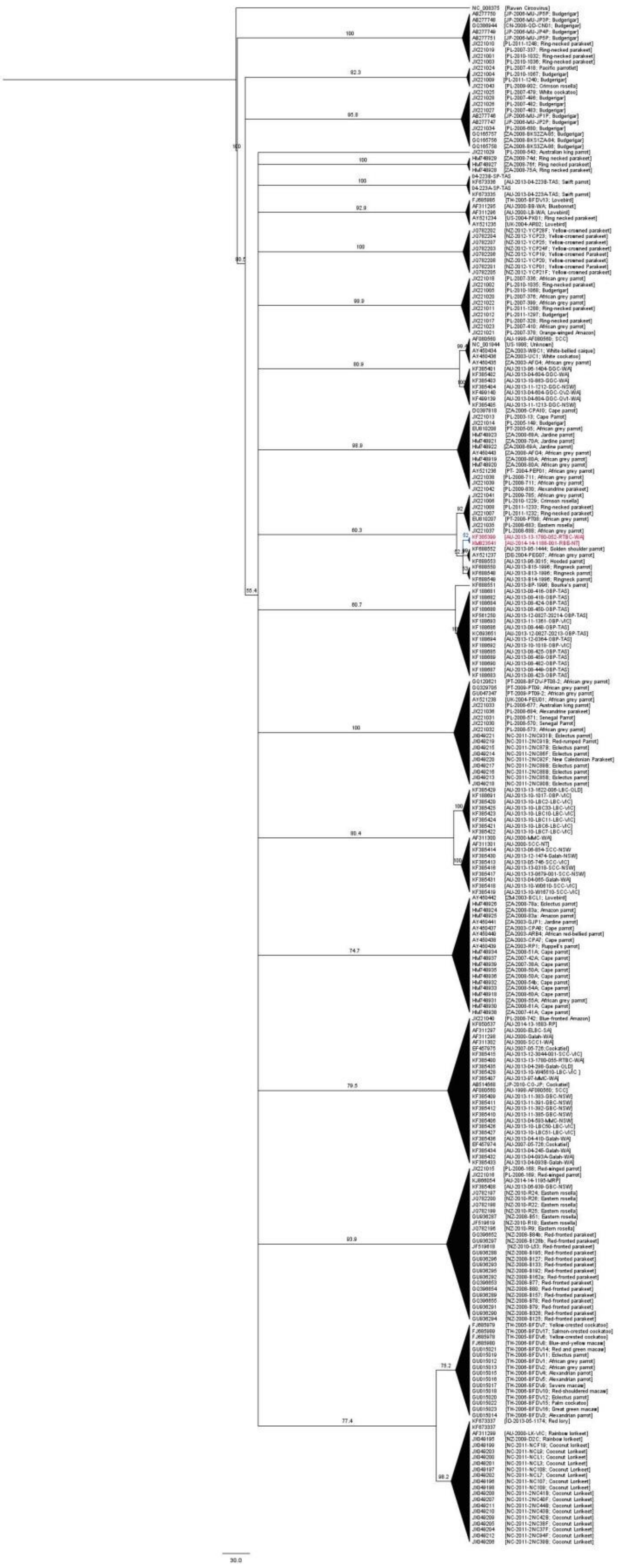

Figure S1. Outgroup-rooted Maximum likelihood (ML) tree for evolutionary relationships among BFDV genomes sequences available in GenBank. The ML tree was constructed using a full-length BFDV genome from a single rainbow bee-eater and all other full-length BFDV genome sequences available on GenBank with 1000 nonparametric bootstrap resamplings. The red text highlighted clade shows the relationship of rainbow bee-eater BFDV with genotypes from a wild red-tailed black cockatoo and a wide variety of parrot species.

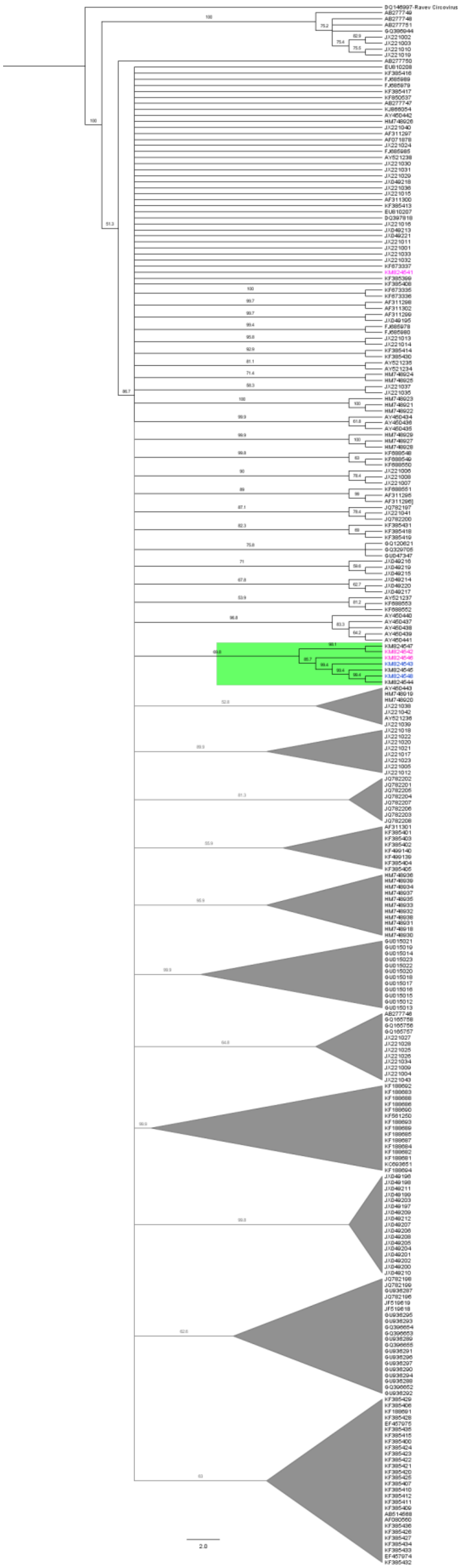

Figure S2. Outgroup-rooted Neighbour-joining (NJ) tree for evolutionary relationships among partial *Rep* gene sequences of BFDV. The NJ tree was constructed using partial *Rep* gene sequences of BFDV from rainbow bee-eaters with other equivalent sequences available on GenBank, using 1000 nonparametric bootstrap resamplings. Green text highlighted clade shows all sequences obtained from rainbow bee-eaters. Red and blue colour texts highlights genotypic variants originated within individual birds 14-1186-02 and 14-1186-04, respectively.

Table S1. Sequences used for discovering evolutionary pathway of BFDV in rainbow bee-eaters

Accession number	Country	Year of Isolation	Common name	Host Species	Wild/Captive	Reference
KM823541	Australia	2014	Rainbow bee-eaters	*Merops ornatus*	Captive	This study
KM823542	Australia	2014	Rainbow bee-eaters	*Merops ornatus*	Captive	This study
KM823543	Australia	2014	Rainbow bee-eaters	*Merops ornatus*	Captive	This study
KM823544	Australia	2014	Rainbow bee-eaters	*Merops ornatus*	Captive	This study
KM823545	Australia	2014	Rainbow bee-eaters	*Merops ornatus*	Captive	This study
KM823546	Australia	2014	Rainbow bee-eaters	*Merops ornatus*	Captive	This study
KM823547	Australia	2014	Rainbow bee-eaters	*Merops ornatus*	Captive	This study
KM823548	Australia	2014	Rainbow bee-eaters	*Merops ornatus*	Captive	This study
KF688548	Australia	2013	Ringneck parrot	*Barnardius zonarius semitorquatus*	Wild	Sarker et al. (unpublished)
KF688549	Australia	2013	Ringneck parrot	*Barnardius zonarius semitorquatus*	Wild	Sarker et al. (unpublished)
KF688550	Australia	2013	Ringneck parrot	*Barnardius zonarius semitorquatus*	Wild	Sarker et al. (unpublished)
KF688551	Australia	2013	Bourke's parrot	Neopsephotus bourkii	Wild	Sarker et al. (unpublished)
KF688552	Australia	2013	Golden shoulder parrot	Psephotus chrysopterygius	Wild	Sarker et al. (unpublished)
KF688553	Australia	2013	Hooded parrot	Psephotus dissimilis	Wild	Sarker et al. (unpublished)
KF673335	Australia	2013	Swift parrot	*Lathamus discolour*	Wild	Sarker et al., 2014
KF673336	Australia	2013	Swift parrot	*Lathamus discolour*	Wild	Sarker et al., 2014
KF673337	Australia	2013	Red lory	*Eos bornea*	Wild	Sarker et al., 2014
KF188681	Australia	2013	Orange-bellied parrot	*Neophema chrysogaster*	Captive	Sarker et al., 2014
KF188682	Australia	2013	Orange-bellied parrot	*Neophema chrysogaster*	Captive	Sarker et al., 2014
KF188683	Australia	2013	Orange-bellied parrot	*Neophema chrysogaster*	Captive	Sarker et al., 2014
KF188684	Australia	2013	Orange-bellied parrot	*Neophema chrysogaster*	Captive	Sarker et al., 2014
KF188685	Australia	2013	Orange-bellied parrot	*Neophema chrysogaster*	Captive	Sarker et al., 2014
KF188686	Australia	2013	Orange-bellied parrot	*Neophema chrysogaster*	Captive	Sarker et al., 2014
KF188687	Australia	2013	Orange-bellied parrot	*Neophema chrysogaster*	Captive	Sarker et al., 2014
KF188688	Australia	2013	Orange-bellied parrot	*Neophema chrysogaster*	Captive	Sarker et al., 2014
KF188689	Australia	2013	Orange-bellied parrot	*Neophema chrysogaster*	Captive	Sarker et al., 2014
KF188690	Australia	2013	Orange-bellied parrot	*Neophema chrysogaster*	Captive	Sarker et al., 2014
KF188691	Australia	2013	Orange-bellied parrot	*Neophema chrysogaster*	Captive	Sarker et al., 2014
KF188692	Australia	2013	Orange-bellied parrot	*Neophema chrysogaster*	Captive	Sarker et al., 2014
KF188693	Australia	2013	Orange-bellied parrot	*Neophema chrysogaster*	Captive	Sarker et al., 2014
KF188694	Australia	2013	Orange-bellied parrot	*Neophema chrysogaster*	Captive	Sarker et al., 2014
KC693651	Australia	2013	Orange-bellied parrot	*Neophema chrysogaster*	Wild	Peters et al., 2014
KF561250	Australia	2013	Orange-bellied parrot	*Neophema chrysogaster*	Wild	Sarker et al., 2014
KF385399	Australia	2013	Red-tailed black cockatoo	*Calyptorhynchus banksii*	Wild	Sarker et al., 2014
KF385400	Australia	2013	Red-tailed black cockatoo	*Calyptorhynchus banksii*	Wild	Sarker et al., 2014
KF385401	Australia	2013	Gang-gang cockatoo	*Callocephalon fimbriatum*	Captive	Sarker et al., 2014
KF385402	Australia	2013	Gang-gang cockatoo	*Callocephalon fimbriatum*	Captive	Sarker et al., 2014
KF385403	Australia	2013	Gang-gang cockatoo	*Callocephalon fimbriatum*	Captive	Sarker et al., 2014
KF385404	Australia	2013	Gang-gang cockatoo	*Callocephalon fimbriatum*	Wild	Sarker et al., 2014
KF385405	Australia	2013	Gang-gang cockatoo	*Callocephalon fimbriatum*	Wild	Sarker et al., 2014
KF385406	Australia	2013	Major Mitchell’s cockatoo	*Lophochroa leadbeateri*	Captive	Sarker et al., 2014
KF385407	Australia	2013	Major Mitchell’s cockatoo	*Lophochroa leadbeateri*	Captive	Sarker et al., 2014
AF311300	Australia	2000	Major Mitchell’s cockatoo	*Lophochroa leadbeateri*	Captive	Bassami et al., 2001
KF385408	Australia	2013	Glossy black cockatoo	*Calyptorhynchus lathami*	Captive	Sarker et al., 2014

KF385409	Australia	2013	Glossy black cockatoo	*Calyptorhynchus lathami*	Captive	Sarker et al., 2014
KF385410	Australia	2013	Glossy black cockatoo	*Calyptorhynchus lathami*	Captive	Sarker et al., 2014
KF385411	Australia	2013	Glossy black cockatoo	*Calyptorhynchus lathami*	Captive	Sarker et al., 2014
KF385412	Australia	2013	Glossy black cockatoo	*Calyptorhynchus lathami*	Captive	Sarker et al., 2014
KF385413	Australia	2013	Sulphur-crested cockatoo	*Cacatua galerita*	Captive	Sarker et al., 2014
KF385414	Australia	2013	Sulphur-crested cockatoo	*Cacatua galerita*	Captive	Sarker et al., 2014
KF385415	Australia	2013	Sulphur-crested cockatoo	*Cacatua galerita*	Captive	Sarker et al., 2014
KF385416	Australia	2013	Sulphur-crested cockatoo	*Cacatua galerita*	Wild	Sarker et al., 2014
KF385417	Australia	2013	Sulphur-crested cockatoo	*Cacatua galerita*	Wild	Sarker et al., 2014
KF385418	Australia	2013	Sulphur-crested cockatoo	*Cacatua galerita*	Wild	Sarker et al., 2014
KF385419	Australia	2013	Sulphur-crested cockatoo	*Cacatua galerita*	Wild	Sarker et al., 2014
AF080560	Australia	1998	Sulphur-crested cockatoo	*Cacatua galerita*	Captive	Bassami et al., 1998
AF311301	Australia	2000	Sulphur-crested cockatoo	*Cacatua galerita*	Wild	Bassami et al., 2001
AF311302	Australia	2000	Sulphur-crested cockatoo	*Cacatua galerita*	Captive	Bassami et al., 2001
AY450436	South Africa	2003	White cockatoo	*Cacatua alba*	Captive	Heath et al., 2004
GU015022	Thailand	2006	Palm cockatoo	*Probosciger aterrimus*	Captive	Sariya et al., (unpublished)
FJ685980	Thailand	2006	Blue-and-yellow macaw	*Ara ararauna*	Captive	Sariya et al., (unpublished)
FJ685978	Thailand	2005	Yellow-crested cockatoo	*Cacatua sulphurea*	Captive	Sariya et al., (unpublished)
FJ685979	Thailand	2005	Yellow-crested cockatoo	*Cacatua sulphurea*	Captive	Sariya et al., (unpublished)
FJ685989	Thailand	2006	Salmon-crested cockatoo	*Cacatua moluccensis*	Captive	Sariya et al., (unpublished)
KF385420	Australia	2013	Long-billed corella	*Cacatua tenuirostris*	Wild	Sarker et al., 2014
KF385421	Australia	2013	Long-billed corella	*Cacatua tenuirostris*	Wild	Sarker et al., 2014
KF385422	Australia	2013	Long-billed corella	*Cacatua tenuirostris*	Wild	Sarker et al., 2014
KF385423	Australia	2013	Long-billed corella	*Cacatua tenuirostris*	Wild	Sarker et al., 2014
KF385424	Australia	2013	Long-billed corella	*Cacatua tenuirostris*	Wild	Sarker et al., 2014
KF385425	Australia	2013	Long-billed corella	*Cacatua tenuirostris*	Wild	Sarker et al., 2014
KF385426	Australia	2013	Long-billed corella	*Cacatua tenuirostris*	Wild	Sarker et al., 2014
KF385427	Australia	2013	Long-billed corella	*Cacatua tenuirostris*	Wild	Sarker et al., 2014
KF385428	Australia	2013	Long-billed corella	*Cacatua tenuirostris*	Wild	Sarker et al., 2014
KF385429	Australia	2013	Long-billed corella	*Cacatua tenuirostris*	Wild	Sarker et al., 2013
AF311297	Australia	2000	Eastern long-billed corella	*Cacatua tenuirostris*	Captive	Bassami et al., 2001
KF385430	Australia	2013	Galah	*Eolophus roseicapillus*	Captive	Sarker et al., 2014
KF385431	Australia	2013	Galah	*Eolophus roseicapillus*	Wild	Sarker et al., 2014
KF385432	Australia	2013	Galah	*Eolophus roseicapillus*	Wild	Sarker et al., 2014
KF385433	Australia	2013	Galah	*Eolophus roseicapillus*	Captive	Sarker et al., 2014
KF385434	Australia	2013	Galah	*Eolophus roseicapillus*	Captive	Sarker et al., 2014
KF385435	Australia	2013	Galah	*Eolophus roseicapillus*	Wild	Sarker et al., 2014
KF385436	Australia	2013	Galah	*Eolophus roseicapillus*	Captive	Sarker et al., 2014
AF311298	Australia	2000	Galah	*Eolophus roseicapillus*	Captive	Bassami et al., 2001
EF457974	Australia	2007	Cockatiel	*Nymphicus hollandicus*	Captive	Shearer et al., 2008
EF457975	Australia	2007	Cockatiel	*Nymphicus hollandicus*	Captive	Shearer et al., 2008
AB514568	Japan	2010	Cockatiel	*Nymphicus hollandicus*	Captive	Kotah et al., 2010
AY450434	South Africa	2003	White-bellied caique	*Pionites leucogaster*	Captive	Heath et al., 2004
AF071878	USA	1998	Unknown (pooled blood)	*Unknown*	Captive	Niagro et al., 1998
NC_001944	USA	1998	Unknown (pooled blood)	*Unknown*	Captive	Niagro et al., 1998
GQ396652	New Zealand	2008	Red-fronted parakeet	*Cyanoramphus novaezelandiae*	Wild	Ortiz-Catedral et al., 2010
GQ396653	New Zealand	2008	Red-fronted parakeet	*Cyanoramphus novaezelandiae*	Wild	Ortiz-Catedral et al., 2010
GQ396656	New Zealand	2008	Red-fronted parakeet	*Cyanoramphus novaezelandiae*	Wild	Ortiz-Catedral et al., 2010

GQ396654	New Zealand	2008	Red-fronted parakeet	*Cyanoramphus novaezelandiae*	Wild	Ortiz-Catedral et al., 2010
GQ396655	New Zealand	2008	Red-fronted parakeet	*Cyanoramphus novaezelandiae*	Wild	Ortiz-Catedral et al., 2010
GU936291	New Zealand	2008	Red-fronted parakeet	*Cyanoramphus novaezelandiae*	Wild	Massaso et al., 2012
GU936292	New Zealand	2008	Red-fronted parakeet	*Cyanoramphus novaezelandiae*	Wild	Massaso et al., 2012
GU936297	New Zealand	2008	Red-fronted parakeet	*Cyanoramphus novaezelandiae*	Wild	Massaso et al., 2012
JF519618	New Zealand	2010	Red-fronted parakeet	*Cyanoramphus novaezelandiae*	Wild	Massaso et al., 2012
GU936296	New Zealand	2008	Red-fronted parakeet	*Cyanoramphus novaezelandiae*	Wild	Massaso et al., 2012
GU936295	New Zealand	2008	Red-fronted parakeet	*Cyanoramphus novaezelandiae*	Wild	Massaso et al., 2012
GU936294	New Zealand	2008	Red-fronted parakeet	*Cyanoramphus novaezelandiae*	Wild	Massaso et al., 2012
GU936290	New Zealand	2008	Red-fronted parakeet	*Cyanoramphus novaezelandiae*	Wild	Massaso et al., 2012
GU936288	New Zealand	2008	Red-fronted parakeet	*Cyanoramphus novaezelandiae*	Wild	Massaso et al., 2012
GU936293	New Zealand	2008	Red-fronted parakeet	*Cyanoramphus novaezelandiae*	Wild	Massaso et al., 2012
GU936289	New Zealand	2008	Red-fronted parakeet	*Cyanoramphus novaezelandiae*	Wild	Massaso et al., 2012
JF519619	New Zealand	2010	Eastern rosella	*Platycercus eximius*	Wild	Massaso et al., 2012
JQ782196	New Zealand	2010	Eastern rosella	*Platycercus eximius*	Wild	Massaso et al., 2012
GU936287	New Zealand	2008	Eastern rosella	*Platycercus eximius*	Wild	Massaso et al., 2012
JQ782198	New Zealand	2010	Eastern rosella	*Platycercus eximius*	Wild	Massaso et al., 2012
JQ782199	New Zealand	2010	Eastern rosella	*Platycercus eximius*	Wild	Massaso et al., 2012
JQ782197	New Zealand	2010	Eastern rosella	*Platycercus eximius*	Wild	Massaso et al., 2012
JQ782200	New Zealand	2010	Eastern rosella	*Platycercus eximius*	Wild	Massaso et al., 2012
AF311295	Australia	2000	Bluebonnet	*Psephotus haematogaster*	Captive	Bassami et al., 2001
AF311296	Australia	2000	Rosey-faced lovebird	*Agapornis roseicollis*	Captive	Bassami et al., 2001
AY521235	United Kingdom	2004	Rosey-faced lovebird	*Agapornis roseicollis*	Captive	de Kloet & de Kloet, 2004
FJ685985	Thailand	2005	Lovebird	*Agapornis sp*	Captive	Sariya et al., (unpublished)
AY450442	Zambia	2003	Black-cheeked lovebird	*Agapornis personata*	Captive	Heath et al., 2004
AY450435	South Africa	2003	African grey parrot	*Psittacus erithacus*	Captive	Heath et al., 2004
GU015023	Thailand	2006	Great green macaw	*Ara ambigua*	Captive	Sariya et al., (unpublished)
GU015020	Thailand	2006	Eclectus parrot	*Eclectus roratus*	Captive	Sariya et al., (unpublished)
GU015014	Thailand	2006	Alexandrian parrot	*Psittacula eupatria*	Captive	Sariya et al., (unpublished)
GU015012	Thailand	2006	African grey parrot	*Psittacus erithacus*	Captive	Sariya et al., (unpublished)
GU015013	Thailand	2006	African grey parrot	*Psittacus erithacus*	Captive	Sariya et al., (unpublished)
GU015018	Thailand	2006	Red-shouldered macaw	*Ara nobilis*	Captive	Sariya et al., (unpublished)
GU015015	Thailand	2006	Alexandrian parrot	*Psittacula eupatria*	Captive	Sariya et al., (unpublished)
GU015016	Thailand	2006	Alexandrian parrot	*Psittacula eupatria*	Captive	Sariya et al., (unpublished)
GU015017	Thailand	2006	Severe macaw	*Ara severa*	Captive	Sariya et al., (unpublished)
GU015021	Thailand	2006	Red and green macaw	*Ara chloropterus*	Captive	Sariya et al., (unpublished)
GU015019	Thailand	2006	Eclectus parrot	*Eclectus roratus*	Captive	Sariya et al., (unpublished)
AY521234	USA	2004	Ring necked parakeet	*Psittacula kramerii*	Captive	de Kloet & de Kloet, 2004
HM748929	South Africa	2008	Ring necked parakeet	*Psittacula krameri*	Captive	Varsani et al., 2011
HM748927	South Africa	2008	Ring necked parakeet	*Psittacula krameri*	Captive	Varsani et al., 2011
HM748928	South Africa	2008	Ring necked parakeet	*Psittacula krameri*	Captive	Varsani et al., 2011
AY450437	South Africa	2003	Cape parrot	*Poicephalus robustus*	Captive	Heath et al., 2004
AY450438	South Africa	2003	Cape parrot	*Poicephalus robustus*	Captive	Heath et al., 2004
AY450439	South Africa	2003	Ruppell's parrot	*Poicephalus rueppelli*	Captive	Heath et al., 2004
AY450440	South Africa	2003	African red-bellied parrot	*Poicephalus rufiventris*	Captive	Heath et al., 2004
DQ397818	South Africa	2006	Cape parrot	*Poicephalus robustus*	Captive	Heath et al., 2004
HM748939	South Africa	2007	Cape parrot	*Poicephalus robustus*	Captive	Varsani et al., 2011
HM748936	South Africa	2007	Cape parrot	*Poicephalus robustus*	Captive	Varsani et al., 2011

HM748935	South Africa	2008	Cape parrot	*Poicephalus robustus*	Captive	Varsani et al., 2011
HM748918	South Africa	2008	Cape parrot	*Poicephalus robustus*	Captive	Varsani et al., 2011
HM748930	South Africa	2008	Cape parrot	*Poicephalus robustus*	Captive	Varsani et al., 2011
HM748938	South Africa	2007	Cape parrot	*Poicephalus robustus*	Captive	Varsani et al., 2011
HM748931	South Africa	2008	African grey parrot	*Psittacus erithacus*	Captive	Varsani et al., 2011
HM748933	South Africa	2008	Cape parrot	*Poicephalus robustus*	Captive	Varsani et al., 2011
HM748932	South Africa	2008	Cape parrot	*Poicephalus robustus*	Captive	Varsani et al., 2011
HM748937	South Africa	2007	Cape parrot	*Poicephalus robustus*	Captive	Varsani et al., 2011
HM748924	South Africa	2008	Amazon parrot	*Amazona sp.*	Captive	Varsani et al., 2011
HM748925	South Africa	2008	Amazon parrot	*Amazona sp.*	Captive	Varsani et al., 2011
HM748926	South Africa	2008	Eclectus parrot	*Eclectus roratus*	Captive	Varsani et al., 2011
JX049197	New Caledonia	2011	Coconut Lorikeet	Trichoglossus haematodus deplanchii	Captive	Julian et al., 2012
JX049198	New Caledonia	2011	Coconut Lorikeet	Trichoglossus haematodus deplanchii	Captive	Julian et al., 2012
JX049200	New Caledonia	2011	Coconut Lorikeet	Trichoglossus haematodus deplanchii	Captive	Julian et al., 2012
JX049201	New Caledonia	2011	Coconut Lorikeet	Trichoglossus haematodus deplanchii	Captive	Julian et al., 2012
JX049202	New Caledonia	2011	Coconut Lorikeet	Trichoglossus haematodus deplanchii	Captive	Julian et al., 2012
JX049203	New Caledonia	2011	Coconut Lorikeet	Trichoglossus haematodus deplanchii	Captive	Julian et al., 2012
JX049205	New Caledonia	2011	Coconut Lorikeet	Trichoglossus haematodus deplanchii	Captive	Julian et al., 2012
JX049206	New Caledonia	2011	Coconut Lorikeet	Trichoglossus haematodus deplanchii	Captive	Julian et al., 2012
JX049207	New Caledonia	2011	Coconut Lorikeet	Trichoglossus haematodus deplanchii	Captive	Julian et al., 2012
JX049208	New Caledonia	2011	Coconut Lorikeet	Trichoglossus haematodus deplanchii	Captive	Julian et al., 2012
JX049209	New Caledonia	2011	Coconut Lorikeet	Trichoglossus haematodus deplanchii	Captive	Julian et al., 2012
JX049210	New Caledonia	2011	Coconut Lorikeet	Trichoglossus haematodus deplanchii	Captive	Julian et al., 2012
JX049211	New Caledonia	2011	Coconut Lorikeet	Trichoglossus haematodus deplanchii	Captive	Julian et al., 2012
JX049212	New Caledonia	2011	Coconut Lorikeet	Trichoglossus haematodus deplanchii	Captive	Julian et al., 2012
JX049213	New Caledonia	2011	Eclectus parrot	*Eclectus roratus*	Captive	Julian et al., 2012
JX049214	New Caledonia	2011	Eclectus parrot	*Eclectus roratus*	Captive	Julian et al., 2012
JX049215	New Caledonia	2011	Eclectus parrot	*Eclectus roratus*	Captive	Julian et al., 2012
JX049216	New Caledonia	2011	Eclectus parrot	*Eclectus roratus*	Captive	Julian et al., 2012
JX049217	New Caledonia	2011	Eclectus parrot	*Eclectus roratus*	Captive	Julian et al., 2012
JX049218	New Caledonia	2011	Eclectus parrot	*Eclectus roratus*	Captive	Julian et al., 2012
JX049219	New Caledonia	2011	Red-rumped Parrot	Psephotus haematonotus	Captive	Julian et al., 2012
JX049220	New Caledonia	2011	New Caledonian Parakeet	Cyanoramphus saisetti	Captive	Julian et al., 2012
JX049221	New Caledonia	2011	Eclectus parrot	*Eclectus roratus*	Captive	Julian et al., 2012
JX049196	New Caledonia	2011	Coconut lorikeet	*Trichoglossus haematodus deplanchii*	Captive	Julian et al., 2012
JX049199	New Caledonia	2011	Coconut lorikeet	*Trichoglossus haematodus deplanchii*	Captive	Julian et al., 2012
JX049204	New Caledonia	2011	Coconut lorikeet	*Trichoglossus haematodus deplanchii*	Captive	Julian et al., 2012
JX049195	Australia	2009	Rainbow lorikeet	*Trichoglossus haematodus*	Captive	Julian et al., 2012
AF311299	Australia	2000	Rainbow lorikeet	*Trichoglossus haematodus*	Captive	Bassami et al., 2001
GQ329705	Portugal	2009	African grey parrot	*Psittacus erithacus*	Captive	Henriques and Fevereiro, (unpublished)
GU047347	Portugal	2009	African grey parrot	*Psittacus erithacus*	Captive	Henriques and Fevereiro, (unpublished)
GQ120621	Portugal	2008	African grey parrot	*Psittacus erithacus*	Captive	Henriques and Fevereiro, (unpublished)
EU810207	Portugal	2008	African grey parrot	*Psittacus erithacus*	Captive	Henriques and Fevereiro, (unpublished)
EU810208	Portugal	2005	African grey parrot	*Psittacus erithacus*	Captive	Henriques and Fevereiro, (unpublished)
AY521237	Germany	2004	African grey parrot	*Psittacus erithacus*	Captive	de Kloet & de Kloet, 2004
AY450443	South Africa	2003	African grey parrot	*Psittacus erithacus*	Captive	Heath et al., 2004
AY521236	Portugal	2004	African grey parrot	*Psittacus erithacus*	Captive	de Kloet & de Kloet, 2004
HM748920	South Africa	2008	African grey parrot	*Psittacus erithacus*	Captive	Varsani et al., 2011

AY450441	South Africa	2003	Jardine parrot	*Poicephalus gulielmi massaicus*	Captive	Heath et al., 2004
HM748919	South Africa	2008	Jardine parrot	*Poicephalus gulielmi massaicus*	Captive	Varsani et al., 2011
HM748921	South Africa	2008	Jardine parrot	*Poicephalus gulielmi massaicus*	Captive	Varsani et al., 2011
HM748922	South Africa	2008	Jardine parrot	*Poicephalus gulielmi massaicus*	Captive	Varsani et al., 2011
HM748923	South Africa	2008	Jardine parrot	*Poicephalus gulielmi massaicus*	Captive	Varsani et al., 2011
GQ165757	South Africa	2008	Budgerigar	*Melopsittacus undulatus*	Captive	Varsani et al., 2010
GQ165758	South Africa	2008	Budgerigar	*Melopsittacus undulatus*	Captive	Varsani et al., 2010
GQ165756	South Africa	2008	Budgerigar	*Melopsittacus undulatus*	Captive	Varsani et al., 2010
AB277747	Japan	2006	Budgerigar	*Melopsittacus undulatus*	Captive	Ogawa et al., 2010
AB277746	Japan	2006	Budgerigar	*Melopsittacus undulatus*	Captive	Ogawa et al., 2010
AB277748	Japan	2006	Budgerigar	*Melopsittacus undulatus*	Captive	Ogawa et al., 2010
AB277749	Japan	2006	Budgerigar	*Melopsittacus undulatus*	Captive	Ogawa et al., 2010
AB277750	Japan	2006	Budgerigar	*Melopsittacus undulatus*	Captive	Ogawa et al., 2010
AB277751	Japan	2006	Budgerigar	*Melopsittacus undulatus*	Captive	Ogawa et al., 2010
GQ386944	China	2008	Budgerigar	*Melopsittacus undulatus*	Captive	Zhuang et al., (unpublished)
AY521234	USA	2004	Ring necked parakeet	*Psittacula kramerii*	Captive	de Kloet & de Kloet, 2004
JQ782201	New Zealand	2012	Yellow-crowned Parakeet	*Cyanoramphus auriceps*	Wild	Massaso et al., 2012
JQ782202	New Zealand	2012	Yellow-crowned Parakeet	*Cyanoramphus auriceps*	Wild	Massaso et al., 2012
JQ782203	New Zealand	2012	Yellow-crowned Parakeet	*Cyanoramphus auriceps*	Wild	Massaso et al., 2012
JQ782204	New Zealand	2012	Yellow-crowned Parakeet	*Cyanoramphus auriceps*	Wild	Massaso et al., 2012
JQ782205	New Zealand	2012	Yellow-crowned Parakeet	*Cyanoramphus auriceps*	Wild	Massaso et al.,2012
JQ782206	New Zealand	2012	Yellow-crowned Parakeet	*Cyanoramphus auriceps*	Wild	Massaso et al.,2012
JQ782207	New Zealand	2012	Yellow-crowned Parakeet	*Cyanoramphus auriceps*	Wild	Massaso et al., 2012
JQ782208	New Zealand	2012	Yellow-crowned Parakeet	*Cyanoramphus auriceps*	Wild	Massaso et al., 2012
JX221001	Poland	2010	Ring-necked parakeet	*Psittacula krameri*	Captive	Julian et al., 2013
JX221002	Poland	2010	Ring-necked parakeet	*Psittacula krameri*	Captive	Julian et al., 2013
JX221003	Poland	2010	Ring-necked parakeet	*Psittacula krameri*	Captive	Julian et al., 2013
JX221004	Poland	2010	Budgerigar	*Melopsittacus undulatus*	Captive	Julian et al., 2013
JX221005	Poland	2010	Budgerigar	*Melopsittacus undulatus*	Captive	Julian et al., 2013
JX221006	Poland	2010	Crimson rosella	*Platycercus elegans*	Captive	Julian et al., 2013
JX221007	Poland	2011	Ring-necked parakeet	*Psittacula krameri*	Captive	Julian et al., 2013
JX221008	Poland	2011	Ring-necked parakeet	*Psittacula krameri*	Captive	Julian et al., 2013
JX221009	Poland	2011	Budgerigar	*Melopsittacus undulatus*	Captive	Julian et al., 2013
JX221010	Poland	2011	Ring-necked parakeet	*Psittacula krameri*	Captive	Julian et al., 2013
JX221011	Poland	2011	Ring-necked parakeet	*Psittacula krameri*	Captive	Julian et al., 2013
JX221012	Poland	2011	Budgerigar	*Melopsittacus undulatus*	Captive	Julian et al., 2013
JX221013	Poland	2003	Cape Parrot	*Poicephalus robustus*	Captive	Julian et al., 2013
JX221014	Poland	2005	Budgerigar	*Melopsittacus undulatus*	Captive	Julian et al., 2013
JX221015	Poland	2006	Red-winged parrot	*Aprosmictus erythropterus*	Captive	Julian et al., 2013
JX221016	Poland	2006	Red-winged parrot	*Aprosmictus erythropterus*	Captive	Julian et al., 2013
JX221017	Poland	2007	Ring-necked parakeet	*Psittacula krameri*	Captive	Julian et al., 2013
JX221018	Poland	2007	African grey parrot	*Psittacus erithacus*	Captive	Julian et al., 2013
JX221019	Poland	2007	Ring-necked parakeet	*Psittacula krameri*	Captive	Julian et al., 2013
JX221020	Poland	2007	African grey parrot	*Psittacus erithacus*	Captive	Julian et al., 2013
JX221021	Poland	2007	Orange-winged Amazon	*Amazona amazonica*	Captive	Julian et al., 2013
JX221022	Poland	2007	African grey parrot	*Psittacus erithacus*	Captive	Julian et al., 2013
JX221023	Poland	2007	African grey parrot	*Psittacus erithacus*	Captive	Julian et al., 2013
JX221024	Poland	2007	Pacific parrotlet	*Forpus coelestis*	Captive	Julian et al., 2013

JX221025	Poland	2007	White cockatoo	*Cacatua alba*	Captive	Julian et al., 2013
JX221026	Poland	2007	Budgerigar	*Melopsittacus undulatus*	Captive	Julian et al., 2013
JX221027	Poland	2007	Budgerigar	*Melopsittacus undulatus*	Captive	Julian et al., 2013
JX221028	Poland	2007	Budgerigar	*Melopsittacus undulatus*	Captive	Julian et al., 2013
JX221029	Poland	2008	Australian king parrot	*Alisterus scapularis*	Captive	Julian et al., 2013
JX221030	Poland	2008	Senegal Parrot	*Poicephalus senegalus*	Captive	Julian et al., 2013
JX221031	Poland	2008	Senegal Parrot	*Poicephalus senegalus*	Captive	Julian et al., 2013
JX221032	Poland	2008	African grey parrot	*Psittacus erithacus*	Captive	Julian et al., 2013
JX221033	Poland	2008	Australian king parrot	*Alisterus scapularis*	Captive	Julian et al., 2013
JX221034	Poland	2008	Budgerigar	*Melopsittacus undulatus*	Captive	Julian et al., 2013
JX221035	Poland	2008	Eastern rosella	*Platycercus eximius*	Captive	Julian et al., 2013
JX221036	Poland	2008	Alexandrine parakeet	Psittacula eupatria	Captive	Julian et al., 2013
JX221037	Poland	2008	African grey parrot	*Psittacus erithacus*	Captive	Julian et al., 2013
JX221038	Poland	2008	African grey parrot	*Psittacus erithacus*	Captive	Julian et al., 2013
JX221039	Poland	2008	African grey parrot	*Psittacus erithacus*	Captive	Julian et al., 2013
JX221040	Poland	2008	Blue-fronted Amazon	*Amazona aestiva*	Captive	Julian et al., 2013
JX221041	Poland	2009	African grey parrot	*Psittacus erithacus*	Captive	Julian et al., 2013
JX221042	Poland	2009	Alexandrine parakeet	*Psittacula eupatria*	Captive	Julian et al., 2013
JX221043	Poland	2009	Crimson rosella	*Platycercus elegans*	Captive	Julian et al., 2013
